# Continuous dry dispersion of multi-walled carbon nanotubes to aerosols with high concentrations of individual fibers

**DOI:** 10.1007/s11051-018-4262-y

**Published:** 2018-06-02

**Authors:** Barbara Katrin Simonow, Daniela Wenzlaff, Asmus Meyer-Plath, Nico Dziurowitz, Carmen Thim, Jana Thiel, Mikolaj Jandy, Sabine Plitzko

**Affiliations:** Federal Institute of Occupational Safety and Health (BAuA), Nöldnerstraße 40 - 42, 10317 Berlin, Germany

**Keywords:** Multi-walled carbon nanotubes, Aerosol generation, Dry powder dispersion, Aerosol morphology

## Abstract

The assessment of the toxicity of airborne nanofibers is an important task. It relies on toxicological inhalation studies and validated exposure measurement techniques. Both require nanofiber-containing aerosols of known morphological composition and controlled fraction of individual fibers. Here, a dry powder dispersion method is presented that operates with mixtures of nanofibers and microscale beads. Aerosolization experiments of mixtures of multi-walled carbon nanotubes (MWCNTs) and glass beads that were continuously fed into a Venturi nozzle enabled high generation rates of aerosols composed of individual and agglomerate nanofiber structures. The aerosol process achieved good stability over more than 2 h with respect to concentration and aerodynamic size distribution. Its operation duration is limited only by the reservoir volume of the cyclone used to separate the beads from the aerosol. The aerosol concentration can be controlled by changing the mass ratio of MWCNTs and glass beads or by adapting the mass feed rate to the nozzle. For two agglomerated MWCNT materials, aerosol concentrations ranged from 1700 to 64,000 nano-objects per cm^3^. Comprehensive scanning electron microscope analysis of filter samples was performed to categorize and determine the morphological composition of the aerosol, its fiber content as well as fiber length and diameter distributions. High fractions of individual fibers of up to 34% were obtained, which shows the setup to be capable of dispersing also highly tangled MWCNT agglomerates effectively.

**Electronic supplementary material:**

The online version of this article (10.1007/s11051-018-4262-y) contains supplementary material, which is available to authorized users.

## Introduction

The unique properties of carbon nanotubes (CNTs) make them a widely studied material with promising commercial applications. The production capacity and use of CNTs have been reported to steadily increase (De Volder et al. 2013). In accord to the fiber toxicology paradigm (Pott and Friedrichs 1972; Stanton and Wrench 1972), their fibrous morphology and biodurability have raised concerns about potential lung carcinogenicity especially for multi-walled CNTs (MWCNTs). While highly tangled MWCNT agglomerates with granular morphology have been reported to show no fiber-toxic effect (Pauluhn 2009), inhalation exposure studies of well-dispersed MWCNTs have observed asbestos-like carcinogenicity (Oberdörster et al. 2015). Mercer et al. (2013) have shown that individual and short MWCNT structures may translocate to secondary organs like the liver. Also mesothelial injury, characteristic for asbestos, was observed for MWCNTs after intraperitoneal injection of well-dispersed CNT suspensions (Nagai et al. 2011; Rittinghausen et al. 2014). The inhalative exposure to individual and fibrous CNT structures is therefore considered a health risk, in particular for individual MWCNTs and CNT bundles with a geometry according to the World Health Organization’s (WHO) fiber definition of a length exceeding 5 μm, a diameter below 3 μm, and an aspect ratio larger than 3:1 (WHO 1985).

However, the assessment of such nanoscale fibrous objects^1^ in air is challenging. None of the direct-reading measurement instruments currently used to assess airborne nano-object exposures is capable of differentiating granular and fibrous morphologies. In addition, their response to nanofibers and low-density CNT agglomerates is mostly unknown. It has been shown that in-depth interpretation of scanning mobility particle sizer (SMPS) and aerodynamic particle sizer (APS) data requires good knowledge of the aerosol morphology when dealing with fiber-containing aerosol (Brockmann and Rader 1990; Chen et al. 2016). Systematic challenging of such instruments with well-characterized fiber-containing aerosols is therefore required to study principle limitations and response characteristics.

For such inhalation toxicological and instrument response studies, purely fiber-containing aerosols with stable concentrations of morphologically uniform and fully de-agglomerated nanofibers are desirable but currently not available. Considerable work is required for the characterization of fiber-containing aerosols. They need to be sampled and analyzed microscopically at high resolution (Chen et al. 2012).

Various methods for CNT aerosol generation have been developed and reported in the past. They are either based on dry dispersion of CNT powders (Fujitani et al. 2009; McKinney et al. 2009; Myojo et al. 2009; Lee et al. 2010; Plitzko et al. 2010; Chen et al. 2012; Vo and Zhuang 2013; O’Shaughnessy et al. 2014; Vo et al. 2014) or on the spraying of liquid CNT suspensions (Jennerjohn et al. 2010; Seto et al. 2010; Ahn et al. 2011; Bahk et al. 2013; Su and Cheng 2014; Wang et al. 2015). Pneumatic spray systems were successfully used to produce individual and well-dispersed MWCNT aerosols in high and stable concentrations (Lee et al. 2010; Ahn et al. 2011; Su and Cheng 2014).

A challenging and time-consuming step for liquid CNT dispersions however is the preparation of homogenous and temporally stable CNT suspensions needed for these methods. To obtain sufficient wettability of CNTs in water and other polar liquids and to promote their dispersibility and stability in the solvent, significant amounts of surfactants have to be added (Ryman-Rasmussen et al. 2009; Pauluhn and Rosenbruch 2015). In addition, high energy liquid dispersion techniques generally must be applied, including ultrasonication (Shvedova et al. 2005; Ahn et al. 2011), high shear force mixing, or mechanical milling (Ryman-Rasmussen et al. 2009; Seto et al. 2010). High energy dispersion techniques may give rise to significant shortening of fibers. Chemical functionalization of CNTs (Han et al. 2010; Bahk et al. 2013; Wang et al. 2015) may increase the degree and stability of CNT suspensions. Such pre-treatments for liquid dispersion may have considerable effects on the physicochemical material properties. As additional drawback, they generally result in aerosols not of pristine but surfactant-coated objects. Therefore, aerosol generation techniques that can start from pristine, chemically and morphologically unmodified CNT material appear desirable.

Dry powder dispersion techniques can avoid the wetting and stabilization problem of liquid-powder processing. They have been successfully applied for the generation aerosols from pristine CNTs. Batch techniques use a single batch of material that is agitated to transfer energy to CNT agglomerates in order to release fibers, fiber fragments, and agglomerates into an air flow. Powder agitation can be applied by slow or rapid oscillating motion using loudspeakers (McKinney et al. 2009; Porter et al. 2010) and so-called vortex (Lee et al. 2010; Dazon et al. 2017) or linear shakers (Spurny et al. 1975; Fujitani et al. 2009; Plitzko et al. 2010). They have been reported to be capable of releasing micro- and nanoscale MWCNT structures and individual fibers. Although stable aerosol concentration was reported to have been generated with some of these batch-style systems (Fujitani et al. 2009; McKinney et al. 2009), perpetual agitation of a powder batch gradually transforms the morphology of the powder. The applied energy may effect both loosening and compactifying CNTs and their agglomerates by agglomerate abrasion and breakup or agglomerate interlocking and fiber entanglement processes, respectively. Batch-style dry techniques may therefore require careful process optimization in order to maintain a perpetually agitated CNT material in stable dusting condition. For some CNT material batches, such conditions may not be achieved. Especially loosely agglomerated MWCNTs may initially release high aerosol concentrations. They however may exhibit a tendency to interlock and compactify with progressing agitation duration, which would result in changes in aerosol concentration and morphology. Both parameters are however important to assess the temporal stability of aerosol generators used for instrument challenging and exposure studies.

This work presents a dry dispersion technique and comprehensive aerosol characterization approach to provide test aerosols that contain morphologically well-characterized high concentrations of individual and agglomerated fibrous structures. The method operates with a continuous flow of fresh CNT material. It does not suffer from aging effects occurring in batch agitating aerosol generators. The flow of fresh material is provided from mixtures of pristine CNTs and microscale beads. The beads not only dilute the CNT material to the desired mass concentration but also transform it to a free-flowing powder mixture. This facilitates controlled continuous feeding to the aerosolization unit. Here, a Venturi nozzle generates high rates of aerosols from the mixture. In the following, the performance of the technique is evaluated for two MWCNT materials of different agglomerate and fiber morphology. The generated aerosols were injected into a home-built exposure chamber that was designed with focus on high spatial aerosol uniformity and a volume flow large enough to supply multiple measurement or exposure devices simultaneously with CNT-containing aerosols.

## Materials and methods

### Materials

**Fig. 1:**
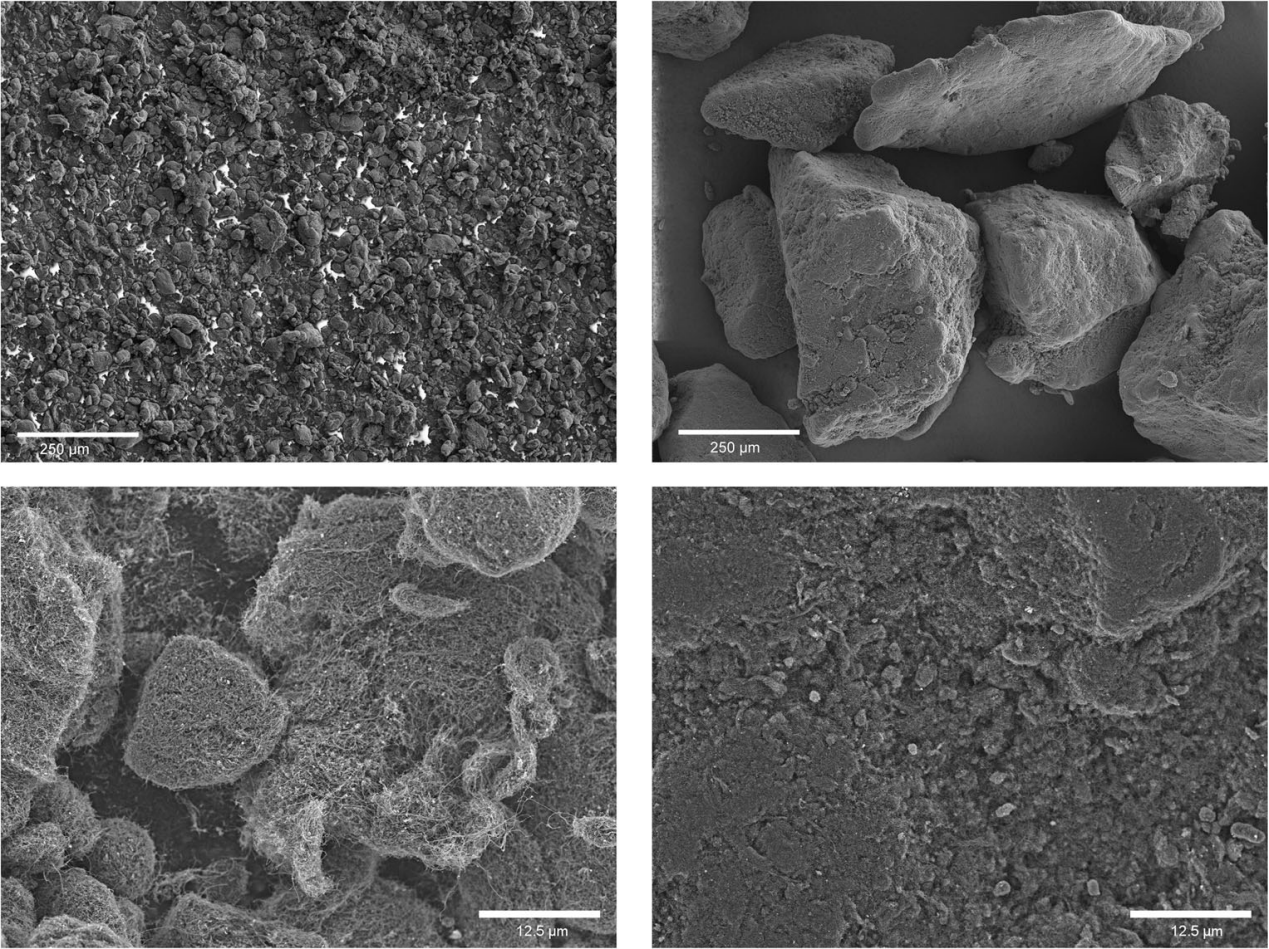
Scanning electron microscope images of the starting material *ARIGM001* (left) and *Baytubes C150P* (right). The lower right image shows the surface of a large agglomerate

In this study, two different types of MWCNT materials were tested: *ARIGM001* (industrial grade; outer diameter (OD) 10–30 nm; length < 15 μm; purity > 80%; Arry International Group Ltd.) and *Baytubes C150P* (95% carbon, OD 13 nm; median length > 1 μm; provided by Bayer Material Science AG), denoted as *Baytubes* in the following. Both were used without prior purification or additional treatment. *Baytubes* were chosen, as they are known to form powders of highly tangled, multi micron-sized granular structure (Pauluhn 2009) and therefore tend to exhibit a low dust release propensity (cf. Fig. 1). Meanwhile, the production of *Baytubes* has been discontinued. The material NM04003a from the JRC Nanomaterial Repository might serve as a substitute (Totaro et al. 2016). SEM images of the starting materials are shown in Fig. 1. TEM images of the two MWCNT materials are included in the supplementary information.

*Swarcoflex* glass beads (GB) (diameter 400–600 μm, roundness ≥ 80%, SWARCO M. Swarovski GmbH) were used as received. They were mixed with the MWCNTs in order to transform the MWCNT powder into a free-flowing powder of reduced MWCNT mass concentration. Prior to CNT aerosolization experiments, the aerosol particle concentration generated by aerosolization of different microscale glass bead types was studied.

For the preparation of mixtures with glass beads, MWCNT powder of a specific mass was added to 1 kg of glass beads in a cylindrical glass bottle of 1 l volume. In this work, the MWCNT concentration of the mixture ranged from 10 mg/kg to 4 g/kg. The MWCNT powder was then mixed with the glass beads by slow revolution of the closed bottle around its axis at about 10–15 rpm. After typically 5 to 15 min of mixing, a visually homogenous, gray colored mixture was obtained.

### Aerosol generation

**Fig. 2:**
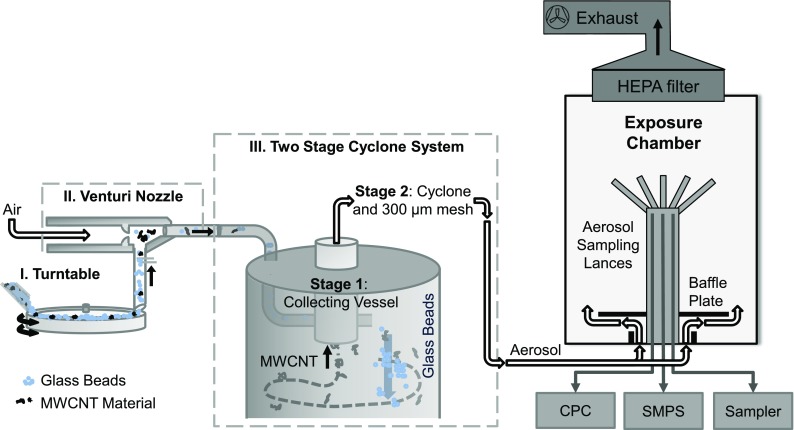
Schematic diagram illustrating the components and working principle of the dry dispersion aerosol generator and its connection to the exposure chamber. A mixture of glass beads and MWCNT material is continuously transported to a Venturi nozzle that aerosoloizes the mixture. Downstream, a two-stage cyclone system separates the airborne fraction from the glass beads and larger MWCNT agglomerates. The aerosol composition in the exposure chamber was monitored and sampled with a set of gas lances, see Supplementary Information

For MWCNT aerosol generation, a dry dispersion system was developed that disperses mixtures of MWCNTs and glass beads by means of a Venturi nozzle. The mixtures were fed volumetrically into a notch in a horizontally rotating turntable. The turntable transported the mixture to the Venturi side inlet, where it was sucked in and was blown through the Venturi nozzle into the feed line. In contrast to shaker-based vibration systems that operate on perpetually agitated batches, the system presented here avoids morphological powder aging effects from perpetual agitation by continuously supplying pristine CNT material to the nozzle. A downstream two-stage cyclone system separated airborne glass beads and larger MWCNT agglomerates from the aerosol fraction that was then introduced to the exposure chamber (see Fig. 2).

A modified SAG 410/U control unit (Topas GmbH, Dresden, Germany) was used to control the rotational speed of the turntable in the range from 8 to 12 rpm as well as the pressure of dry and particle free air that was fed into the primary Venturi nozzle inlet at 4–5 bar. The MWCNT/GB mixture was poured from the reservoir bottle into a stainless steel funnel. Its opening was adjusted directly above the notch of 10-mm width and 5-mm depth, cut into the upper surface of the turntable. The MWCNT/GB mixture flowing into this notch was continuously transported towards the side inlet of a Venturi nozzle (ISO 5011) with an inlet nozzle diameter of 0.7 mm and an outlet flow of 15–20 L/min. The aerosolized mixture was fed into a home-built two-stage cyclone system consisting of a metal container of 5 L volume, 8.5 cm diameter, and 22.8 cm height as first cyclone stage. A modified FSP 2 sampling head (DEHA Haan & Wittmer GmbH, Heimsheim, Germany) with integrated SIMPEDS cyclone (Harris and Maguire 1968) served as second cyclone stage. A 300 μm stainless steel filter mesh in the outlet of the first cyclone prevented coarser objects to enter the second stage filter in case of cyclone overload. The tangential inlet of 8 mm diameter and the central riser pipe outlet of 20 mm diameter and 60 mm length of the first cyclone very effectively removed the majority of glass beads and larger CNT agglomerates from the aerosolized powder mixture. The secondary cyclone served to further narrow down the object size distribution.

During operation, the feeding rate of the MWCNT/GB mixture into the nozzle was controlled via the speed of the turntable. Continuous weighting of the first cyclone’s metal container enabled mass-controlled feeding. For the present work, feeding rates of 10–13 g/min of MWCNT/GB mixtures were used.

The use of several bottles and larger storage containers for the CNT/GB mixture enables long-term operation of the generator. For the experiments presented here, for materials safety reasons, the metal container of the first cyclone had no revolving door outlet as is commonly used for industrial cyclones and allows for emptying the cyclone during operation. Therefore, the operation time of our experiments was limited to about 3 h to prevent overload of the first cyclone at a mass of about 2.5 kg glass beads.

### Instrumentation

Experimental details of the chamber used for the aerosolization and aerosol homogeneity studies are given in the Supplementary Information.

The particle number concentration in the exposure chamber was monitored with a Grimm CPC model 5.403 (Grimm Aerosol GmbH, Ainring, Germany), operating with a sampling flow rate of 0.3 L/min. Measurement of the electrical mobility size distribution in the range of 10 to 1,100 nm was performed with a Grimm Scanning Mobility Particle Sizer (SMPS) consisting of a CPC model 5.403 and an electrostatic differential mobility analyzer (DMA) of “Vienna” type model L-DMA. The SMPS was likewise operating with a sampling flow rate of 0.3 L/min at a scanning cycle time of 7 min. SMPS data analysis was done with the software provided by the manufacturer (Grimm Software 5.477). Large objects with aerodynamic diameters from 0.5–20 μm were detected with an Aerodynamic Particle Sizer (APS) model TSI 3321 (TSI GmbH, Aachen, Germany) equipped with a 100:1 diluting stage model TSI 3302A. The APS size distribution samples were taken with scanning times of 59 s at a sampling flow rate of 5 L/min.

For studies of morphology distributions, aerosols were sampled on track etch membrane filters (gold-coated polycarbonate with 37-mm diameter and a pore size of nominal 200 nm supplied by APC GmbH, Eschborn, Germany, using PGP sampler units (DEHA Haan & Wittmer GmbH, Heimsheim, Germany). Depending on aerosol concentration, sampling times of 5–60 min and flow rates of 2–3 L/min were applied.

All samplers and measurement instruments were connected to stainless steel sampling gas lances of the chamber using antistatic silicone tubes supplied by Grimm Aerosol GmbH (Asbach et al. 2016).

### Experimental procedure

Before the start of an experiment, the exposure chamber was flushed with dried and HEPA-14-filtered compressed air until a number concentration of 0–60 particles/cm^3^ and a relative humidity of 20–26% was reached inside of the chamber. Next, the aerosol generator was connected to the aerosol inlet of the chamber and the air flow to the Venturi nozzle inlet was adjusted. After the start of the turntable, the MWCNT/GB mixture was poured into the funnel of the aerosol generator to initiate aerosol generation (*t* = 0). For the present setup, a maximum of 2.5 kg MWCNT/GB could be aerosolized continuously. At the standard air flow rate of 20 L/min, completely flushing the 400 L exposure chamber with aerosol required about 20 min. This is reflected in the temporal development visible in Fig. 6. Plateau concentration values were not reached before about 30 min of operation. All mean concentration values given in the following were obtained at minimum 30 min after start of operation.

All silicone tubes used for chamber and instrument connections were cleaned or replaced after approximately 3–5 experiments. Prior to injecting a new type of MWCNT material, the inner surfaces of the exposure chamber and all measurement lances were cleaned and all silicone tubes were replaced to avoid cross contamination.

### Determination of the system's background concentration

**Fig. 3:**
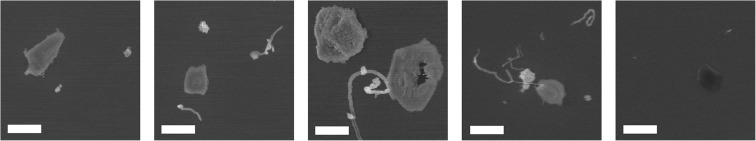
Images of background particles originating from glass bead lot No. 058 during dispersion of mixtures with *ARIGM001*. The particles were collected on a silicon wafer by electrostatic precipitation. The white scale bar has a length of 500 nm

The background concentration arising from the use of glass beads for the aerosolization process was determined for each delivered glass bead lot of about 50 kg mass. For this purpose, 3 kg of pure glass beads was aerosolized with the aerosol generator at a feeding rate of 12 to 13 g/min. The resulting particle number concentration in the exposure chamber was monitored over time. After typically 30 min, a concentration plateau was reached and maintained as long as the glass beads were aerosolized. For the present study, the glass bead lot No. 058 was used. It reached a median plateau concentration of (1143 ± 185) particles/cm^3^, according to CPC monitoring, averaged from minute 30 to 120. As shown in Fig. 3, the background particles were non-fibrous, compact particles of relatively low SEM image contrast. They were easily distinguishable from fibrous MWCNT particles during morphological analysis.

### Electron microscopic analysis of sampled aerosols

Analyses of aerosols sampled on track etch membrane filters were performed with a scanning electron microscope (SEM, Hitachi SU8030, Hitachi High-Technologies Europe GmbH, Krefeld, Germany). A central area of the filter was imaged at a magnification of ×3000, an accelerating voltage of 3 kV and a working distance of 6.1 nm resulting in a minimum feature detection size of 8.3 nm (edge size of a pixel). For each sample, images with a standard resolution of 5120 × 3840 pixels and 1344 μm^2^ area were acquired at 10 to 15 randomly chosen filter positions and were subjected to subsequent morphological characterization.

**Fig. 4:**
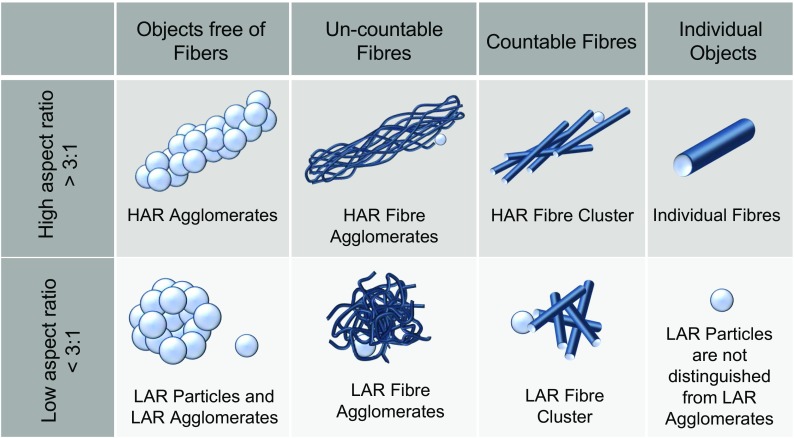
Illustration of the seven object categories used to categorize the SEM imaged objects by their visual morphology, their structure, and their degree of agglomeration

All objects imaged by SEM were categorized visually according to their shape, structure, and degree of agglomeration into one of the seven object categories shown in Fig. 4. These categories do not cover all possible aerosol morphologies. They were chosen according to the subject of the present study that addresses the dispersion state of fibrous and agglomerated fibrous materials as generated by our setup. We therefore differentiated between objects with aspect ratios greater (high aspect ratio, HAR) or smaller (low aspect ratio, LAR) than 3:1 as well as between objects with or without visual fibrous structures. For objects with fibrous structures, we further distinguished between individual fibers, weakly bounded agglomerates with a countable number of elements, named clusters, and highly agglomerated structures with an uncountable number of elements, named agglomerates in the following. Particulate, non-fibrous objects were not distinguished with respect to their agglomeration state. Fibers and fiber-containing agglomerates attached to granular objects were categorized as fibrous objects since attaching catalyst and catalyst support particles are a common phenomenon for industrial grade CNT materials.

The image manipulation software GIMP (version 2.8.6, GNU Image Manipulation Program) was used to visually detect and count all objects found on SEM images in accordance with their category. GIMP’s path tool together with an in-house developed “script-fu” plugin was used to measure the geometrical length of individual fibers by drawing a path along the fiber and converting its length in pixels to nanometers using the image resolution.

## Results and discussion

### Mixing of MWCNTs with glass beads

To test the performance of the dry dispersion aerosolization system, mixtures were prepared of glass beads with either pristine *ARIGM001* or pristine *Baytubes* MWCNTs in different mass ratios (see Table 1). Horizontal rotations of the cylindrical mixing bottle around its rotational axis for a few minutes achieved an apparently homogenous distribution of the MWCNT material in the glass beads. However, as the variations of the temporal stability of the aerosols generated from a continuous material in Fig. 6 suggest, the homogeneity achieved by such a mixing approach cannot be assessed with high accuracy by visual inspection alone. In some batches, MWCNT-enriched or depleted zones appear to have survived in the mixture. In future experiments, the use of alternative, e.g., wobble mixing techniques, and of lifting blades inside of the mixing bottle should be studied to further improve the mixing homogeneity.

**Table 1:** Overview of the prepared mixtures of MWCNTs and glass beads together with their experimental parameters for aerosolization

Experiment	MWCNT material	MWCNT mass fraction^a^ [g/kg]	Experimental parameters for aerosolization		
			Nozzle feeding rate [g/min]	Nozzle outlet flow [L/min]	Mean object number concentration^b^ [#/cm^3^]
A10	ARIGM001	0.01	11.0	16.9	4000 ± 320
A11	ARIGM001	0.01	12.0	19.8	3600 ± 130
A12	ARIGM001	0.01	13.9	19.9	2900 ± 175
A20	ARIGM001	0.06	14.7	19.9	6600 ± 580
A21	ARIGM001	0.06	10.3	19.8	8200 ± 90
A30	ARIGM001	0.20	12.4	19.8	52,000 ± 2300
A31	ARIGM001	0.20	9.7	19.8	46,000 ± 2200
A40	ARIGM001	0.33	11.0	16.9	64,000 ± 8200
B10	Baytubes	0.40	10.5	19.6	1700 ± 130
B20	Baytubes	4.00	10.5	19.6	7500 ± 1300

^a^MWCNT mass per glass bead mass in the mixture

^b^Determined 30 min after start. Standard derivation given

Close-up visual inspection also revealed small-scale local inhomogeneities in the mixture caused by larger MWCNT agglomerates. They survived even if rotational mixing durations exceeded 1 h. This suggests that ball milling effects of the glass beads did not effectively micronize all MWCNT agglomerates. This was especially the case for *Baytubes* MWCNTs, which were synthesized in highly tangled form and large agglomerates (see Fig. 1). Such local inhomogeneities could introduce short-term fluctuations of the aerosol concentration that might not be significant in large exposure chambers.

**Fig. 5:**
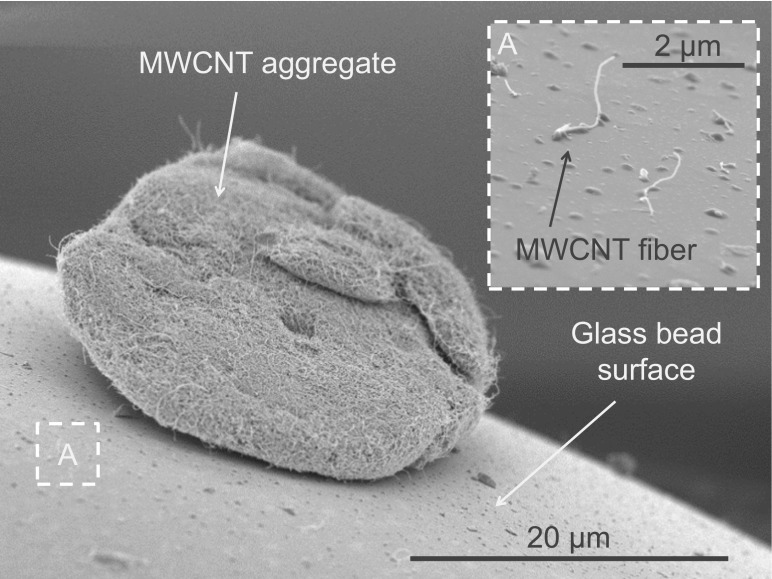
SEM image of MWCNTs attaching to a glass bead surface found in a mixture of ARIGM001 and glass beads. The sample was coated with an iridium layer of 4-nm thickness prior to SEM analysis

The miscibility and transport behavior of MWCNTs and glass beads may be promoted by attractive interaction between glass and MWCNTs. Such attraction may result from van-der-Waals interaction (dispersion forces) and from electrostatic forces. Dispersion forces are governed by the contact area between two object surfaces and are material independent (Hamaker 1937), whereas triboelectrical charge separation can lead to strong electrostatic interaction depending on material pairing (Matsusaka et al. 2010). If at least one partner is an electrical insulator, separated surface charges of opposite sign cannot recombine easily if created by friction (tribocharging) between to two materials of differing electronegativity. Glass is known to exhibit a strong triboelectrically electropositive character, whereas the CNTs may serve as electron donor partner. SEM analysis of a glass bead mixture with *ARIGM001* showed that single MWCNT fibers and even agglomerates adhere to glass bead surfaces (see Fig. 5). Other bead materials with different triboelectrical potential, surface roughness, or polar and dispersive surface energy components may show different nanotube adherence performance. Other interesting bead materials could be, e.g., stainless steel, zirconia, or polymers like polystyrene or polytetrafluoroethylene. Higher surface roughness beads may promote not only adherence to the bead but also CNT abrasion from agglomerate surfaces during mixing. The optimum strength of interaction forces and the optimum material pairing are presently unclear. Excessive adherence of individual fibers should be avoided since it may lower the concentration of fiber morphologies in the aerosol.

A benefit of dry dispersion of MWCNTs in glass beads is the long-term storability of dry powder mixtures, since, different to liquid dispersions, no sedimentation of MWCNTs among glass beads occurs. Using a protective inert gas atmosphere may reduce possible oxidative material aging during mixing and storage in a gas-tight bottle.

### Temporal development of the aerosol concentration

**Fig. 6:**
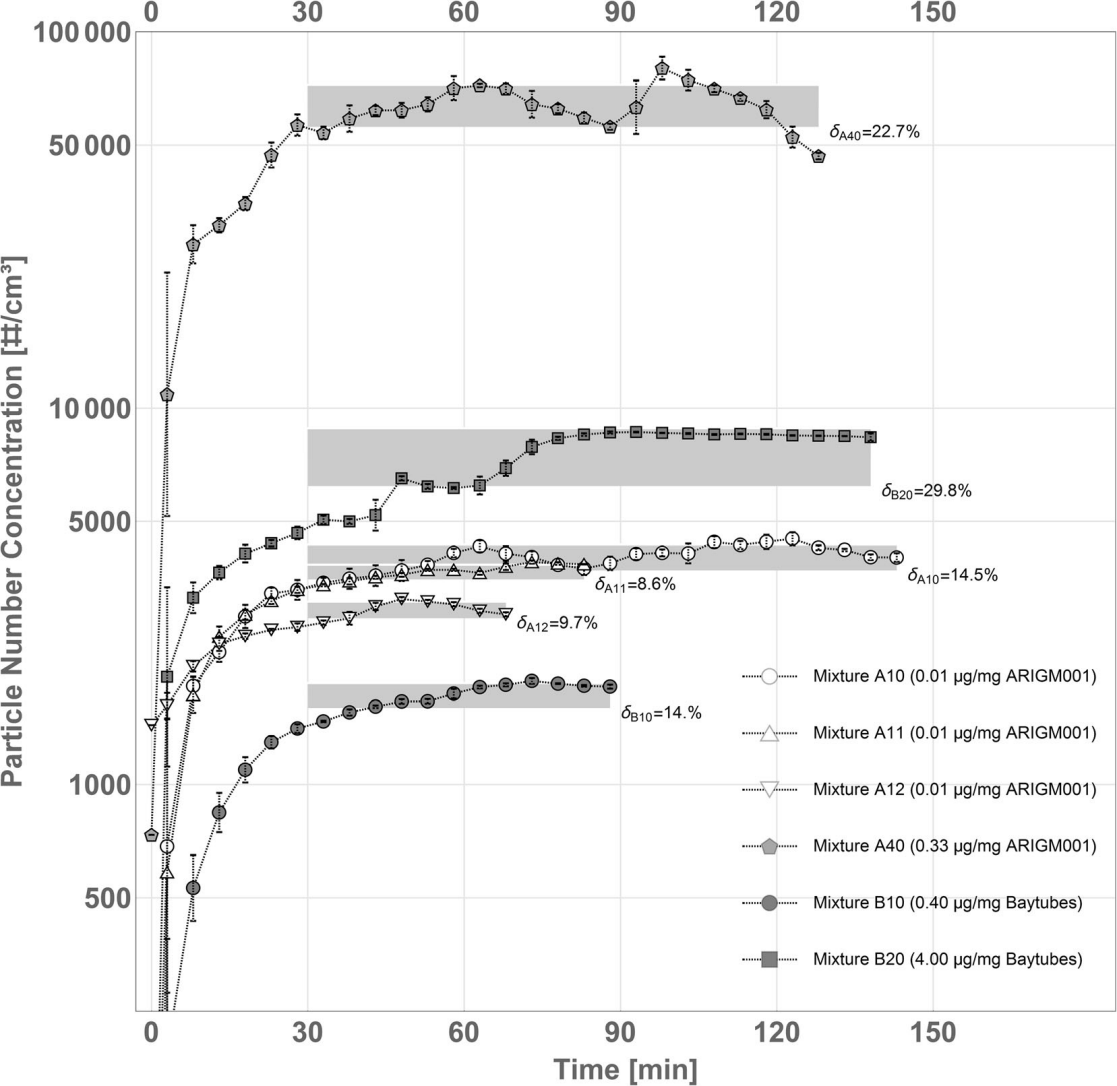
Temporal development of the concentration of MWCNT aerosols, as measured by CPC, during dry dispersion aerosolization experiments, cf. Table 1. Gray areas indicate the 1-sigma confidence level of the data for integration over the total experiment time starting from minute 30. The relative errors for this timespan are given as ***δ*** values in the diagram

The aerosol concentration generated with the studied aerosolization system was monitored with a CPC over time. Figure 6 shows the results for the experiments A11, A12, A13, A40, B10, and B20, which were containing mixtures of glass beads and MWCNTs either of type pristine *ARIGM001* (“A” experiments) or of type *Baytubes* (“B” experiments) in concentrations as specified in Table 1. Once the feeding of mixtures was started (*t* ≔ 0), the number concentration in the chamber was increasing on a time scale of about 20 min, corresponding to the filling time of a volume of 400 L at an aerosol flow of 20 L/min. After about 30 min, which is about 1.5× the approximate filling time, sufficiently stable concentration levels were observed for most tested MWCNT/GB mixtures. In dependence of the studied MWCNT material and mass ratio in the mixture, mean object number concentrations from (1,723 ± 134) to (64,033 ± 8,209) #/cm^3^ were measured for the plateau region. These concentration levels were maintained as long as the MWCNT/GB mixture was continuously transported to the Venturi nozzle. In our experiments, we were able to produce MWCNT aerosols for up to 140 min, before aerosolization had to be stopped to empty the collecting reservoir of the first cyclone stage. Longer aerosolization times are possible if larger reservoir volumes are used.

At comparable mixture mass feed rate of about 11 g/min, the concentration of MWCNT material in the glass bead mixture had a significant influence on the object number concentration in the resulting aerosols, which can be seen by comparing the results for the mixtures A40 and A10, containing *ARIGM001* with 0.33 and 0.01 g/kg, respectively. The higher concentrated mixture A40 produced about 15 times higher aerosol concentration than mixture A10. A similar trend can be seen for the high and the low concentrated *Baytubes* mixtures B10 and B20, respectively, even though the difference in object number concentration is less pronounced.

It became evident that the output concentration was strongly dependent on the type of MWCNT material: Mixtures with *Baytubes* formed significantly lower aerosol concentrations than those with *ARIGM001* with respect to the specific MWCNT mass feed rate. *Baytubes* were synthesized in large agglomerates of highly tangled structure (see Fig. 1). They most likely require higher energies for break up and detangling into aerosolizable fragments than the smaller and more loosely agglomerated material structure of *ARIGM001*.

The results show that our aerosolization method allows controlling the object number output concentrations by changing the MWCNT mass fraction in the glass bead mixture. This is comparable to atomizing differently concentrated liquid CNT suspensions.

### Temporal development of the aerodynamic size distribution

**Fig. 7:**
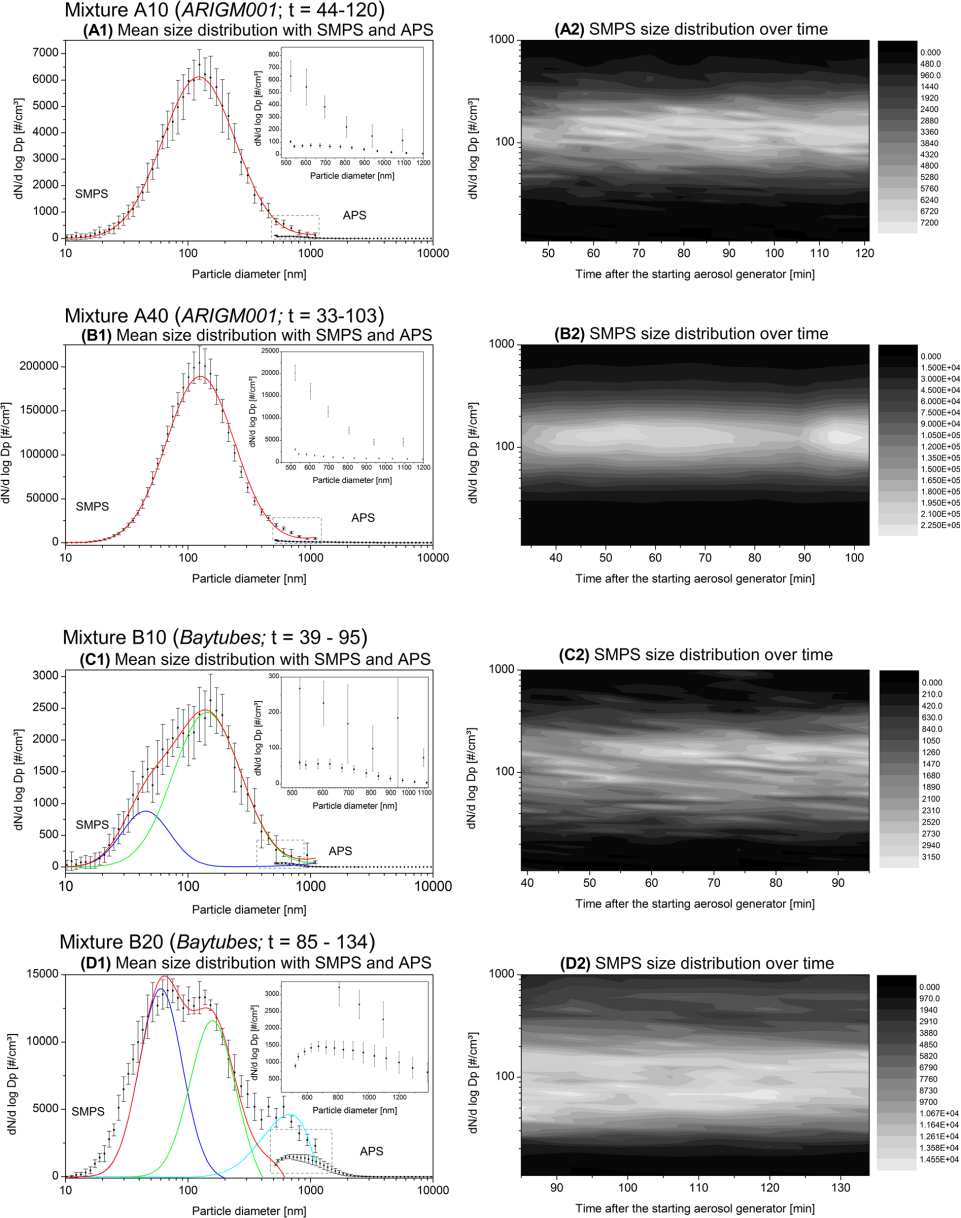
Mean log-normal SMPS and APS size distributions of the MWCNT aerosols from mixture A10, A40 and B10, B20 as measured during time ranges of sufficiently stable number concentrations (*left*, A1–D1). The x-y-z plots on the right show the SMPS size distributions for the same time ranges of all aerosols as function of time (*right*, A2–D2)

**Fig. 8:**
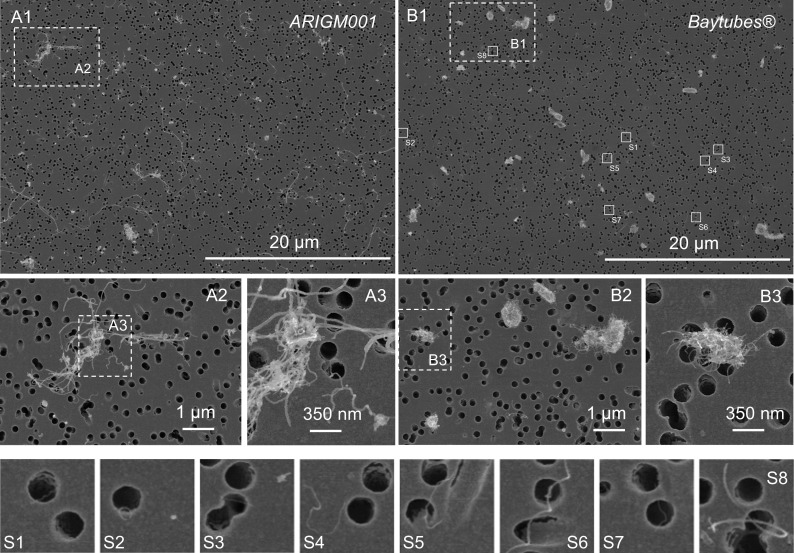
SEM images of collected *ARIGM001* (*left*) and *Baytubes* (*right*) aerosol with magnified areas A1–A3 and B1–B3. Both aerosols were well dispersed and contained of a high fraction out of individual fibers. The insets S1–S7 were added since *Baytubes* exhibit a mean tube diameter of about 14 nm. Therefore individual nanofibers are not easily identified in the upper right group of images B1–B3

Parallel to number concentration monitoring with CPC, size distributions of generated aerosols were monitored with SMPS and APS over time. Figure 7(A1–D1) shows the mean log-normal SMPS and APS size distribution of each aerosol, averaged over several scans and measured in time ranges of sufficiently stable output concentration. For *ARIGM001* aerosols, unimodal size distributions with comparable geometrical mean diameters of 126 nm and respectively 122 nm were measured (Fig. 7(A1–B1)). A broader multimodal size distribution was found for both *Baytubes* aerosols (Fig. 7(C1–D1)). The distributions of *Baytubes* aerosols exhibit a tail towards larger aerodynamic diameters. It indicates that, especially at this high mass concentration of 4 g/kg, *Baytubes* were far less effectively dispersed than *ARIGM001* mixtures. SEM images of the sampled aerosol in Fig. 8 confirm the presence of more microscale agglomerates for *Baytubes* than for *ARIGM001*. Interestingly, the APS size distributions in the range of 500 to 1100 nm were significant lower than those measured with SMPS. A comparable disparity in measurements was reported from Baron et al. (2008) for SWCNT aerosols. The authors assumed that the partly uncertain charging and light absorption behavior of these low-density, open structured, and conductive objects may be the reason for such an observation.

Within the studied time ranges, no significant changes in the size distributions of the MWCNT aerosols were monitored, as can be seen in Fig. 7(A2–D2) showing the SMPS size distributions as a function of time. Although small fluctuations in the total number concentration had an influence on the signal intensities, geometrical mean diameters as well as the distribution width were found to lie in similar ranges for the compared aerosols. This is particularly apparent for the unimodal size distribution of the higher concentrated *ARIGM001* aerosol in Fig. 7(B2). Here, the SMPS size distribution was monitored for 70 min without showing significant changes in distribution width and mean aerodynamic diameter.

The dry dispersion aerosolization system studied here has the potential to produce nanofiber aerosols of highly stable output with respect to both concentration and size distribution for arbitrary times of operation. We believe that the powder mixture feeding system is the key to long-term aerosol stability. It continuously supplies fresh nanofiber material to the Venturi nozzle, which prevents aging effects occurring in perpetually agitated powder beds, but requires the mixture of nanofibers and beads to be sufficiently homogenous. A feedback loop that monitors the concentration in the aerosol supply line and controls the feeding rate via the turntable speed could correct for nanofiber concentration inhomogeneities in the bead mixture.

### Morphological characterization of the MWCNT aerosols by SEM analysis

The generated aerosols were sampled on tracketch membrane filters. SEM images of the filters, as shown representatively in Fig. 8 for an *ARIGM001* and a *Baytubes* aerosol, were used to categorize all observed aerosol objects into one of the seven object categories of Fig. 4. For each sample, between 1000 and 3000 objects were analyzed to characterize the aerosol morphology and to determine the concentration of individual fibers. The results of this categorization are summarized in Table 2. For both *ARIGM001* samples (A10 and A40) as well as for the *Baytubes* sample B20, more than 30% of the collected aerosol objects were individual MWCNT fibers. Moreover, almost 50% of all objects on these samples had a high aspect ratio equal or greater than 3:1. Only for experiment B10 lower ratios of HAR objects and individual fibers were found. This was most probably caused by the ratio of CNT-related and glass bead-related contributions to the aerosol. At the low aerosol concentration of experiment B10, the finite glass bead background became significant: LAR objects increased to about 60%. These findings reveal the necessity to further reduce the background contribution from the glass beads.

**Table 2:** Results of the morphological categorization for the samples A10, A40 and B10, B20. All SEM imaged particles were categorized by their visual shape, structure and estimated degree of agglomeration into 7 particle categories

Percentage of particle category for the sample Total number of the categorized particles	A10 [%] *N* = 3355	A40 [%] *N* = 3327	B10 [%] *N* = 1110	B20 [%] *N* = 2147
HAR particle agglomerates	3.7	2.8	1.3	1.8
HAR fiber agglomerates	4.4	5.2	1.8	6.6
HAR fiber clusters	6.6	9.0	4.1	4.8
Individual fibers	32.1	30.9	21.2	34.5
LAR particle agglomerates and individual particle objects	49.5	44.6	62.1	40.1
LAR fibers agglomerates	3.1	5.1	7.0	9.0
LAR fiber clusters	0.7	2.5	2.5	3.2
Total HAR objects	46.7	47.8	28.4	47.7
Total LAR objects	53.3	52.2	71.6	52.3

The morphological distinction between HAR and LAR objects was introduced here since the aerosol generation method is intended to be used for systematic laboratory studies on nanoparticle instrument response to CNT-containing aerosols of known morphological distribution. As most nanoparticle instruments are designed to count and characterize low aspect ratio particles, it appeared therefore important to determine the amount of individual nanofibers and HAR objects in a test aerosol for the interpretation of instrument responses. Possible limitations of the data obtained from the categorization approach applied here may arise from missing information on the diameter and size of the categorized objects. This prevents estimating mass concentrations from SEM images. In addition, the thin and rather short *Baytubes* fibers found on samples B10 and B20 were categorized the same way as much thicker and longer *ARIGM001* fibers on sample A10 and A40. However, the toxicological relevance of the two fiber morphologies may be quite different to micron-sized powder structures (Pauluhn 2009). Occupational hygiene control may therefore require detecting and distinguishing individual and agglomerate fiber morphologies. It will be a challenge of future research and development to substitute the very laborious task of detecting and categorizing nanofibers on aerosol filter samples, which was performed here, by an *online* fiber detection technique.

The approach followed in the present work gave valuable insights into the HAR object release propensity of two different MWCNTs types under studied dispersion conditions. The very similar individual fiber release ratio came quite unexpected and shows that the applied dispersion energies were high enough to break individual fibers off the surface of agglomerates and to split up larger agglomerates. Such breaking of tangled fibers during dispersion shifts the fiber length distribution to smaller values. However, due to the highly tangled state of the starting materials, the length distributions before and after dispersion cannot be compared, since the lengths of tangled nanotubes cannot be measured reliably in the agglomerated state.

**Fig. 9:**
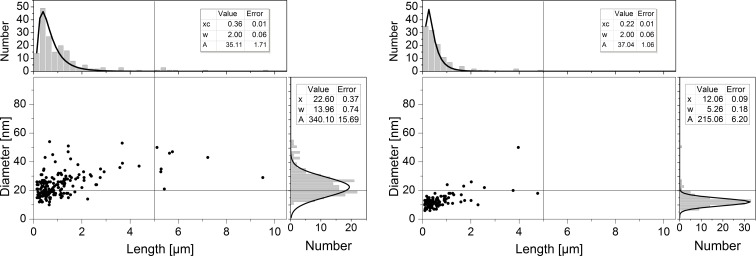
Scatter plots and histograms of the geometrical fiber diameter and length data pairs of individual fibers found in the aerosol sample A10 for *ARIGM001* (*left*, *N* = 176) and aerosol sample B20 for *Baytubes* (*right*, *N* = 118) with log-normal (length) and Gaussian (diameter) peak fitting

For further aerosol characterization of the sampled MWCNT aerosols, the geometrical length and diameter of all imaged and categorized fibers on sample A10 and B20 were determined and plotted as scatter plot and histograms in Fig. 9. Log-normal peak fitting of the geometric fiber length distributions gave an average length of around 350 nm for *ARIGM001* (parameter *w* in Fig. 9), whereas *Baytubes* had a shorter length of about 200 nm. The majority of analyzed MWCNTs (> 90%) exhibited a length below 5 μm. Especially *Baytubes* formed predominantly short fibers below 1.5 μm. This small length is believed to be related to the large agglomerate size and high degree of tanglement of *Baytubes* (cf. Fig. 1). Tangled and interlocked fibers must to be broken to be released from the fiber network and to become observable as individual fibers. The results show the fiber length distribution in the aerosol to be depending on the MWCNT material. Further experiments with other types of CNT materials are recommended to study weather this aerosolization technique is suited for the generation of narrower length or size distributions. Also variations of the air flow rate through the Venturi nozzle that governs dispersive forces should be studied in future.

The aerosolization efficiency (aerosol mass released vs. CNT mass used) of our dry dispersion technique could not be determined here. The mass concentration of the generated nanoscale aerosols was too low to be determined by weighing. A second approach to estimating the aerosol mass based on particle size distribution data obtained from SMPS and APS is not reliable for fibrous aerosols. Firstly, since the main peak(s) of the SMPS size distributions in our experiments resulted from individual fibers but do not provide fiber length information and therefore do not provide mass information. Secondly, for A10, A40, and B10, the contribution of microscale agglomerates in the right hand tail of the SMPS and APS size distributions is insignificant in our data especially for ARIGM001, see insets in Fig. 7(A1–D1). It is nonetheless expected to dominate the total aerosol mass since microscale agglomerates contain many tangled CNTs. For experiment B20, the strong discrepancy between the SMPS and APS reading gives rise to uncertainties on the shape of the size distribution of agglomerates. A third possible approach to estimating the aerosol mass could rely on measuring the volume of agglomerates and the length and diameter of individual fibers imaged with SEM. This is a very laborious task and requires assuming agglomerate densities. Such density assumption is necessary for any mass estimation approach based on agglomerate geometric or mobility diameter data. For partially dispersed CNT materials, these densities are unknown. Future experiments will aim at characterizing aerosols using a so-called nano-particle mass classifier (nano-PMC) that was developed recently (Broßell et al. 2015). In combination with a differential mass analyzer, it allows measuring the mass and density of aerosol particles.

### Reproducibility of the aerosolization technique in replicate experiments

**Fig. 10:**
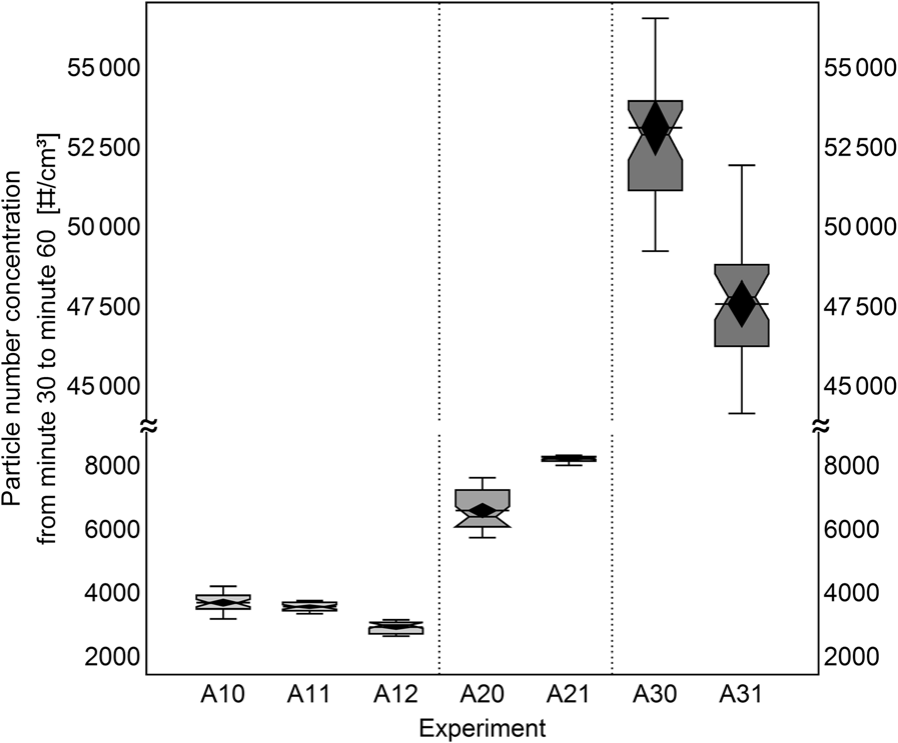
Box-whisker plot for the temporal development of the concentrations generated from *ARIGM001* and glass bead mixtures during minute 30 to 60 after start of feeding (box 25/75%; whisker 1.5; mean: diamond, median: notch). The vertical separators group the three compared mixtures of 0.01, 0.06, or 0.20 g/kg mass ratio MWCNT/GB

The aerosolization technique developed here was also studied with respect to reproducibility of the generated concentration. For this comparison, all *ARIGM001* mixtures were aerosolized under similar experimental conditions. The resulting aerosol number concentrations measured from minute 30 to 60 after start of powder feeding are compared statistically using box-whisker plotting in Fig. 10.

For the evaluated time span, the aerosols of each group of MWCNT/GB mixture with 0.01, 0.06, and 0.20 g/kg in Fig. 10 showed a fair to good degree of reproducibility. For mixtures with equal MWCNT amount, aerosols with mean object number concentrations in the same size range resulted. A maximum standard derivation of 25% in mean object number was found. A feedback loop that monitors the concentration in the aerosol supply line and controls the feeding rate via the turntable speed could correct for CNT inhomogeneities in the glass bead mixture and further improve temporal stability and reproducibility.

### Analogies between dry and liquid aerosolization

It is instructive to review principle analogies between the dry dispersion concept presented here and atomizing concepts using liquid dispersions. It shows that similar components and process steps are required both for dry and liquid dispersion. All of them require individual optimization for best aerosol performance.

The microscale beads used here correspond to a cheap and non-toxic solvent in liquid suspensions. Like a solvent, the beads allow nanofiber material dilution and concentration control. For many material variants, spherical beads can achieve transforming sticky and clogging low-density nanofiber materials into free-flowing powder mixtures. Their liquid-like character facilitates continuous feeding to the atomizing unit, e.g., a Venturi nozzle, and controlling the mass feed rate.

Obtaining homogenous mixtures of nanofiber materials in beads may require optimization of mixing movements which are generally accompanied by ball milling effects on nanofiber agglomerates. The resulting energy transfer to the nanofiber materials is in analogy with, e.g., ultrasonication of nanofibers in liquid suspension. Successful and temporally stable mixing results of nanofibers and beads however do not require pre-treating of the nanofibers with chemical functionalization or surfactant coating as in liquid dispersion.

Even the polarity of a solvent, which is highly critical for preparing liquid suspensions, bears analogies to dry mixing: Optimum triboelectrical pairing of powder components might result in an adhering nanofiber corona on the bead surface and further stabilize nanofibers in the bead mixture via attractive electrostatic forces (Matsusaka et al. 2010).

Finally, the bead-filtering cyclone stage used here is an analogue of a drying unit necessary for obtaining dry aerosols from atomized liquid dispersion.

## Conclusion and perspective

Providing fiber aerosols of controlled individual fiber concentration for fiber toxicological inhalation studies as well as for assessing aerosol measurement performance and exposure limit control strategies is an important task.

Here, a dry dispersion aerosolization technique was developed that uses mixtures of nanofiber materials and microscale beads that are fed continuously through a Venturi nozzle to generate nanofiber-containing aerosols. The technique was studied for two morphologically very different MWCNT materials in glass bead mixtures of varied mass ratio. Aerosols were characterized by CPC, SMPS, APS, and morphological analysis of filter samples with SEM to determine and categorize the morphological composition of the generated aerosols, relative and absolute concentrations of individual fibers as well as fiber length and diameter distributions.

The generator allows controlling the aerosol concentration in an extremely wide range. Aerosols of up to 2 × 10^10^ individual nanofibers per m^3^ were generated at a flow rate of 20 L/min. The obtained MWCNT aerosols contained a high fraction of individual fibers of up to 34%. The toxicological relevance of the obtained fiber aerosols was assessed by quantifying to the content of fibers of WHO geometry. The thicker and more loosely agglomerated material *ARIGM001* resulted in a higher content of WHO fibers in the aerosol than *Baytubes*, both in relative and absolute concentration.

Compared to liquid dispersion and aerosolization of CNTs, dry mixing with glass bead exhibits a number of benefits. It requires neither chemical nor mechanical pre-treatment of the CNTs. Since dry mixtures do not sediment, no possibly toxic organic solvents and surfactants are required to achieve stable dispersions in glass beads. Glass beads are an inexpensive dilution material and may be re-used. Similar to dryer columns for liquid atomization, also our dry dispersion technique requires an additional cyclone stage for downstream separation of glass beads and CNTs. The dry aerosolization process was found to allow high aerosol stability. Observed concentration fluctuations were attributed to partially inhomogeneous mixing of CNTs and glass beads, a process step that requires further improvement.

Future work should be devoted to

- Wind-sifting glass beads prior to use in order to reduce the glass fragment concentration.
- Studying triboelectric charge separation during mixing and its role in attaching nanoscale fragments to beads.
- Optimizing dry mixing to further improve the intended mixing with glass bead and to control ball milling effects of MWCNTs.
- Improving process stability via a feedback loop of the CPC concentration to the powder feeding rate.
- Estimating the energy transfer necessary to breakup or disentangle CNT agglomerates.
- Varying the air flow rate through the Venturi nozzle that governs dispersive forces.
- Further narrowing of the morphological distribution of the aerosol by the use of less tangled materials.

## Electronic supplementary material

ESM 1 (PDF 540 kb)

## Footnotes

1 The term “object” will be used in the following when both granular and fibrous particles are addressed.
