# Supplementary material for: Continuous dry dispersion of multi-walled carbon nanotubes to aerosols with high concentrations of individual fibers
Source: J Nanopart Res. 2018 Jun 2;20(6):154. doi: 10.1007/s11051-018-4262-y (PMC5984959; doi:10.1007/s11051-018-4262-y)
Supplement: Supplementary file 1 — (PDF 540 kb) [file 11051_2018_4262_MOESM1_ESM.pdf]

Continuous dry dispersion of multi-walled carbon nanotubes to aerosols with high concentrations of individual fibers

Barbara Katrin Simonow · Daniela Wenzlaff · Nico Dziurowitz · Carmen Thim · Jana Thiel · Mikolaj Jandy · Asmus Meyer-Plath* · Sabine Plitzko
Federal Institute of Occupational Safety and Health (BAuA), Nöldnerstraße 40 - 42, 10317 Berlin, Germany
*e-mail: meyer-plath.asmus@baua.bund.de

Supplementary Information

SI – Material characterization

Transmission electron microscope images of the two multi-walled carbon nanotube (MWCNT) materials are shown in Figure I, respectively. Both materials exhibit a central bore, characteristic for MWCNTs.

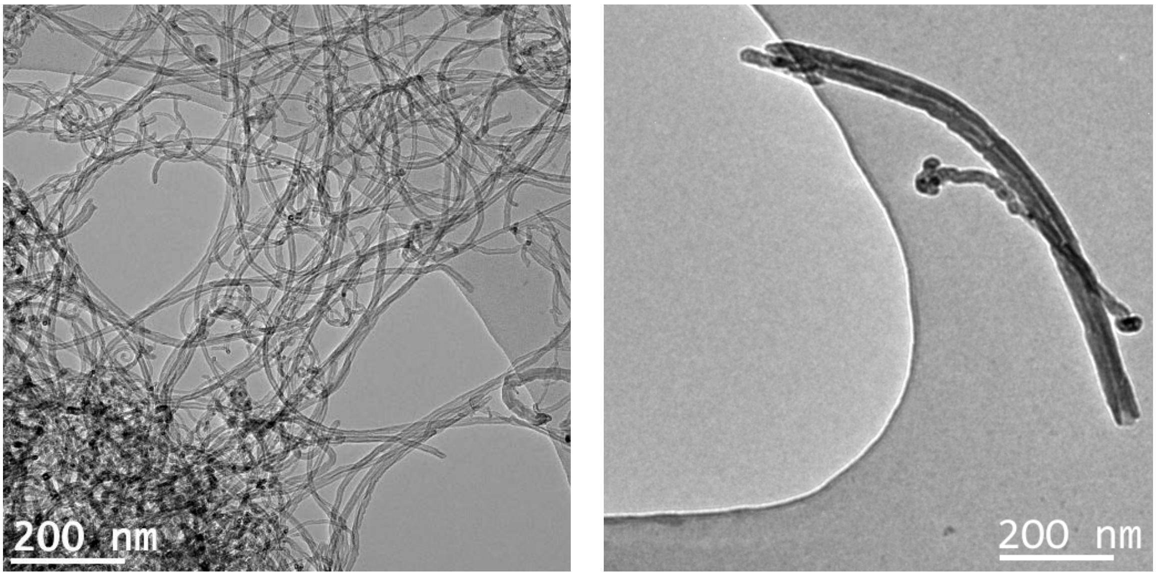

Figure I: Transmission electron microscope images of Baytubes C150P (left) and Arry ARIGM001 (right)

SI – Experimental details of the exposure chamber

The exposure chamber was designed with focus on high spatial aerosol uniformity and on occupational safety aspects related to handling of fiber materials. It consists of a vertically-oriented poly(methyl methacrylate) cylinder 130 cm high and of 63.4 cm inner diameter with about 400 L total volume, maximized with respect to the available space in our laboratory. To suppress locally varying electrostatic wall charging, the chamber's inner polymer surface was equipped with a self-adhesive anti-statically coated polyethylene terephthalate foil that was electrically grounded. The rotational symmetry of the elongated, vertical chamber was selected to promote vertical and radial aerosol uniformity by diffusion of the rising aerosol flow. The upper end of the chamber column was connected via a circular orifice of 42.5 cm diameter to an exhaust line equipped with a filter of HEPA 14 class. The applied exhaust air flow of nominal 120 m³/h caused a negative pressure of about 1.7 – 4.8 mbar inside of the chamber and prevents accidental release of fiber-containing aerosols. The lower bottom flange of the chamber can be lowered to access the inner chamber. It is equipped with the aerosol inlet, four extraction lance feedthroughs and a central column to mount additional extraction lances and measurement equipment. Aerosols were supplied from the aerosol generator through a single tube that was symmetrically split up into two tubes which again were split into two tubes. The resulting four aerosol supply lines were connected to the four quad-

rants of a common ring-shaped volume inside of an aluminium cylinder located at the centre of the bottom chamber flange. The channel volume opened into a narrow horizontal annular slit that was designed to establish tangential uniformity of the ejected aerosol. The radially ejected aerosol was then guided beneath by a circular baffle plate of 40 cm diameter, see Figure II. The baffle plate forced the aerosol to radially spread and then to form a ring-shaped aerosol flow that vertically rose along the outer perimeter of the chamber. This initially radially highly non-uniform, ring-shaped aerosol flow design successfully generates an aerosol region of high radial and tangential uniformity at about mid column height. The underlying concept makes use of the diffusion of fresh aerosol from the outer perimeter of the chamber to the aerosol-depleted central region of the chamber that was shielded from direct aerosol supply by the central baffle plate. Figure II also shows gas streamlines that illustrate the aerosol homogenization concept of using a baffle plate and cylindrical chamber symmetry. The stream lines were predicted qualitatively from numerically solving the Navier-Stokes equation in cylinder coordinates with boundary conditions set by the chamber geometry, see next section.

SI – Experimental details on the determination of the spatial homogeneity of aerosols

The spatial distribution of the particle number concentration of the aerosol was measured by means of a swivelling sampling lance. It was rotatable around its off-axis outer chamber perimeter (Figure IV) and adjustable in height. This way, not all lateral chamber coordinates were reachable but only those at the swivel radius of the bent lance's tip. However, a large vertical range was assessable by adjusting the lance height relative to the chamber's bottom flange and to measure at different rotation angles. In order to deduce radial concentration information from this swivel dataset, the tangential aerosol uniformity of the aerosol was checked by means of the 8-fold star-shaped gas lance setup at the head of the central column shown in Figure II. The 8 gas lances sampled at a fixed radius of 22 cm.

The radial geometry of a particle-free gas flow inside of the chamber was simulated by numerically solving the Navier-Stokes partial differential equation in cylinder coordinates for radial and vertical gas velocity and pressure on a finite element mesh approximating to the chamber geometry. Dirichlet boundary conditions defined the radial inflow velocity at the annular inlet slit and the outlet pressure as well as no-flow conditions on the chamber walls. The numerical solution was obtained and visualised using the software Mathematica 11 by Wolfram Research.

Performance of the experimental setup with respect to spatial homogeneity was studied on the background particle concentration of glass beads (lot No. 058) that were aerosolized at 12 g/min and 20 L/min airflow. In a series of measurements, the first CPC was connected to the first of the 8 gas lances of the 8-fold star-shaped gas lance setup at the head of the central column (Figure II). A second CPC successively measured the aerosol concentrations at the other 7 gas lances. The agreement between the concentration at the first and at all other angular locations was found to be better than 5 %. These findings together with the mapping measurement of the aerosol distribution with the swivelling, height adjusted lance (Figure IV) allowed concluding that at our operation conditions a volume region formed in the chamber where the aerosol distribution exhibited good spatial uniformity. The results are shown in Figure III. For an aerosol flow of 20 L/min, this homogeneous region was located between 60 and 80 cm chamber column height and extended radially to about 27 cm from the central axis. The structure of the observed inhomogeneities in Figure II resembles the overlaid stream lines that resulted from the numerical solution of the Navier-Stokes equation for a particle-free gas flow. It illustrates how the initially ring-shaped flow profile develops into a flattened-top flow profile between 60 and 80 cm height.

A vertical measurement of the aerosol concentration profile inside of the chamber in Figure IV suggests that about 30 % of the aerosol particles are lost between 29 and 80 cm air column height. These losses may be attributed to losses on the outer chamber wall.

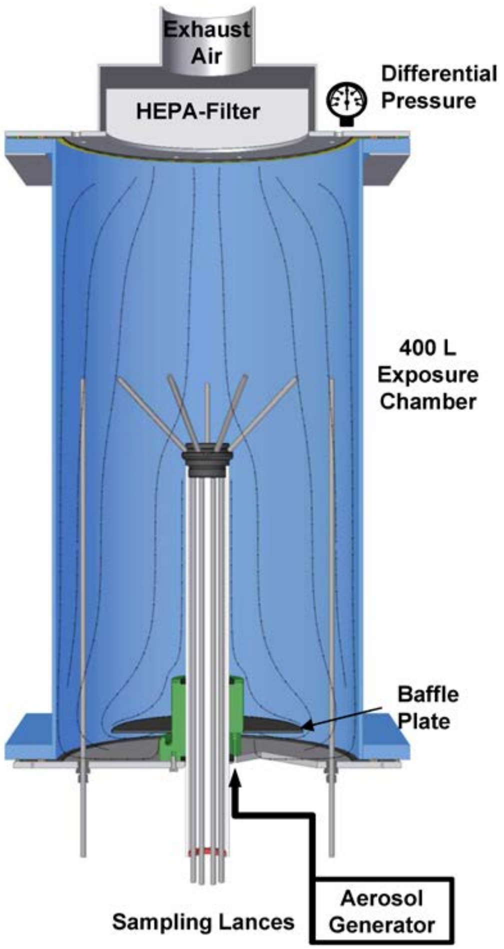

Figure II: Schematic diagram of the 400 L exposure chamber equipped with 12 sampling lances, 8 of them at the central column. Also shown are gas streamlines that illustrate the aerosol homogenization concept of using a baffle plate and cylindrical chamber symmetry

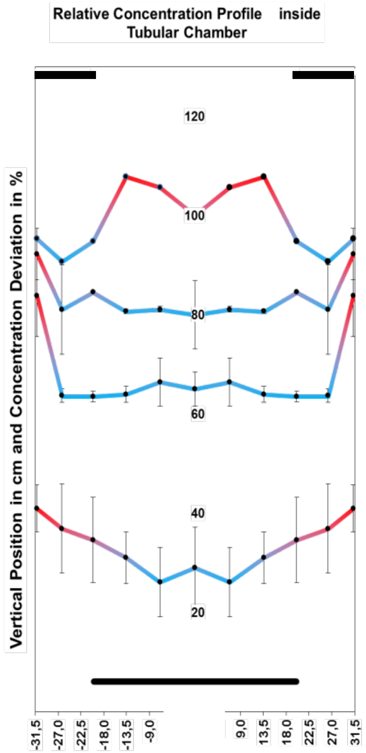

Figure III: Measurement results of vertical and radial relative aerosol concentrations in the chamber at 20 L/min flow rate using glass beads and the swivelling sampling lance (see text). The relative radial concentration curves were placed at their corresponding vertical position (in cm). The concentrations are given in % relative to the central value at r=0. The numeric scale has to be interpreted as a relative one, e.g., the data sets at 30 cm and 90 cm height that range from 26-45 and 90-108, exhibit a span of 19% and 18%, respectively

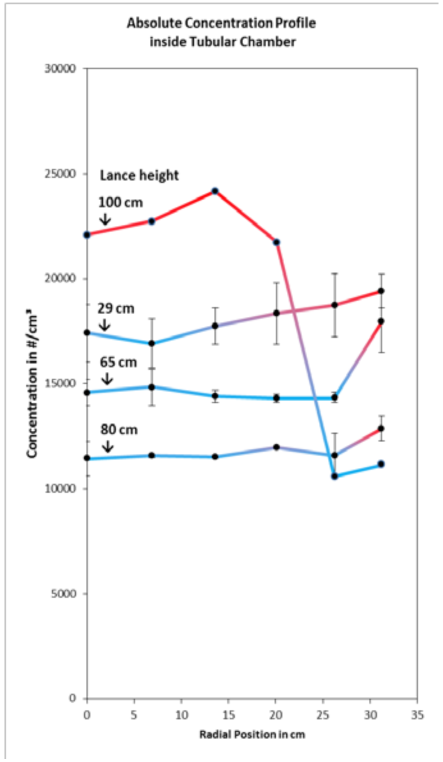

Figure IV: Measurement results of vertical and radial absolute aerosol concentrations in the chamber at 20 L/min flow rate using glass beads and the swivelling sampling lance (see text). The absolute radial concentration curves correspond to the given vertical position in cm. The concentrations are given in #/cm³ according to the left axis scale

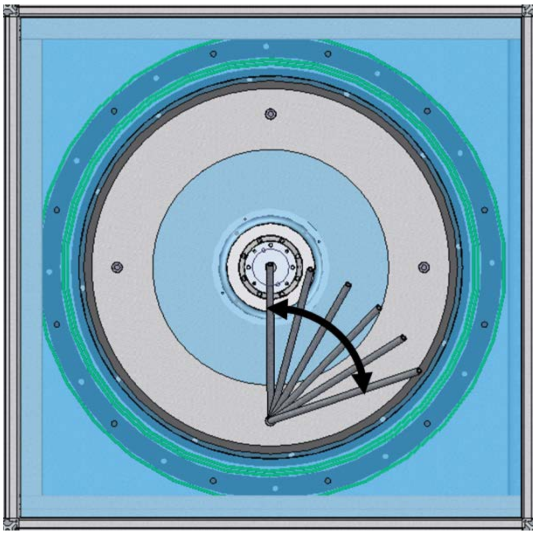

Figure V: Schematic of the movement of the swivelling gas lance used for vertical and radial uniformity studies. The lance was swivels in 15° steps over the full accessible range from -75° to +75°
